# Previous COVID-19 infection significantly reduces elastase levels in newly diagnosed pulmonary tuberculosis patients

**DOI:** 10.3389/fimmu.2025.1586789

**Published:** 2025-07-31

**Authors:** Nancy Hilda Joseph, Alangudi Palaniappan Natarajan, Saravanan Natarajan, Chinnaiyan Ponnuraja, Madeshwaran A, Gunaparvathy I, Hemalatha P, Rajeshwari S, Lavanya Jayabal, Mahilmaran Ayyamperumal, Ramesh P. M., Luke Elizabeth Hanna

**Affiliations:** ^1^ Department of Virology & Biotechnology, Indian Council of Medical Research- National Institute for Research in Tuberculosis, Chennai, India; ^2^ Department of Biochemistry, Indian Council of Medical Research (ICMR)- National Institute for Research in Tuberculosis, Chennai, India; ^3^ Department of Statistics, Indian Council of Medical Research- National Institute for Research in Tuberculosis, Chennai, India; ^4^ Government Hospital of Thoracic Medicine, Tambaram, Chennai, India; ^5^ Institute of Thoracic Medicine, Chennai, India; ^6^ National Tuberculosis Elimination Programme (NTEP), Chennai, India; ^7^ Government Thiruvatteeswarar Hospital of Thoracic Medicine, Chennai, India

**Keywords:** neutrophil extracellular traps, elastase, diabetes, pulmonary tuberculosis, X-ray

## Abstract

**Introduction:**

Tuberculosis (TB) is considered a risk factor for severe COVID-19 disease and the quality of life of patients co-infected with COVID-19 and TB is significantly impacted due to the nature of these diseases. It is still unknown how our immune system will respond to both these pathogens in sequel. As it has been discovered that Neutrophil extracellular traps (NETs) result in caseating granulomas in TB and pathology in COVID-19, we conducted this work to determine the amounts of NET molecules in the bloodstream and to comprehend their function during TB and subsequent SARS-CoV-2 infection.

**Methods:**

We recruited 43 healthy volunteers, 40 newly diagnosed pulmonary tuberculosis patients who were negative for SARS-CoV-2 IgG antibody and 18 newly diagnosed pulmonary tuberculosis patients who were positive for SARS-CoV-2 IgG.

**Results:**

Although Citrullinated Histone H3 and myeloperoxidase, did not show any difference in their levels, the NET marker elastase had significantly reduced circulatory levels in the tuberculosis group with SARS-CoV IgG positivity compared to tuberculosis group without SARS-CoV-2 IgG positivity.

**Discussion:**

The substantial decrease in elastase levels observed in the diabetic cohort of TB patients with SARS-CoV-2 IgG positivity is intriguing and needs large cohort studies in the future to understand the influence of diabetes in TB patients exposed to SARS-CoV-2.

## 1 Introduction

Globally, tuberculosis (TB) is the leading cause of death from a single infectious pathogen (1). The COVID-19 pandemic had caused disruptions to health services worldwide but the globe is slowly revamping to its original form. Regarding TB, the pandemic increased mortality from TB co-infection in addition to its well-documented negative effects on TB case reporting and accessibility to TB-related care (2). Both TB and COVID-19 are contagious infections of the respiratory system that present with comparable symptoms (3, 4). *Mycobacterium tuberculosis* (Mtb) infection is found to increase the susceptibility to SARS-CoV-2 and COVID-19 severity (5). Elderly TB patients have been known to rapidly acquire a severe form of COVID-19, which can cause heightened inflammatory responses and a lengthy recovery period (6); Furthermore, COVID-19 patients have reduced frequency of Mtb–specific CD4^+^ T cells, with possible implications for TB disease progression (7). This demonstrates that Mtb infection complicates the pathogenesis of COVID-19 and vice versa, representing a bidirectional aggravation of disease severity. Meta-analyses have shown that simultaneous diagnosis of COVID-19 and TB increases mortality (8).

We would be better able to treat and prevent co-infection if we had a clear understanding of how our immune system eliminates both of these infections and/or how the pathogen avoids the host’s immune system. Even though different aspects of the immune response to COVID-19 (9) and TB (10) have been well studied, it is unclear how our immune system reacts to both these pathogens in sequel. Neutrophil extracellular trap (NET) formation contributes to a number of SARS-CoV-2 infection sequelae through inflammatory responses and infiltration (11). Acute lung injury (ALI), acute respiratory distress syndrome (ARDS), pulmonary thrombosis and multi-organ damage (MOD) in severe COVID-19 (12) are known to be caused by NETs. In Pulmonary Tuberculosis (PTB), although the release of NETs has been documented (13), it is unclear how they influence the host defense against collateral damage or protective immunity. Our aim is to investigate NET levels in a condition where the immune e system is actively responding to individuals with a history of prior exposure to SARS-CoV-2. This is based on the evidence that NETs contribute to COVID-19 pathogenesis, where their role in TB remains poorly defined. We also investigated the correlation between NET markers, hematological, radiological, and biochemical parameters under the studied conditions.

## 2 Methods

### 2.1 Participants and blood collection

**Figure 1:**
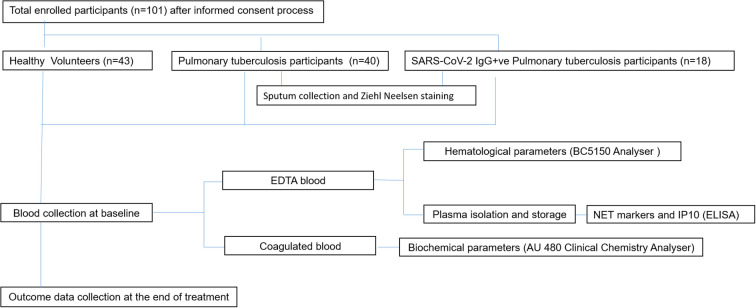
An overview of the whole study is presented in the given figure. The workflow started from participant screening followed by participant recruitment based on predetermined inclusion/exclusion criteria. Specimen collection was done after obtaining written informed consent [Healthy (n=43), PTB (n=40), SARS-CoV-2 IgG+ve PTB (n=18)]. EDTA treated blood and coagulated blood were used for measuring hematological and biochemical parameters respectively using appropriate instruments. Plasma NET markers and IP10 were measured through ELISA. The results were then analysed and interpreted using appropriate statistical methods.

Institutional ethical committee’s (IEC) clearance was obtained from ICMR-National Institute for Research in Tuberculosis (IEC No: 2020 038). The study included three groups: Healthy volunteers (HV) (N =43), newly diagnosed pulmonary tuberculosis patients (PTB) without a documented record of COVID-19 infection (N=40) and PTB participants with a documented record of COVID-19 infection (SARS-CoV-2 IgG+ve PTB) (N=18). Newly diagnosed adult PTB patients in the age group of 18–60 yrs [within three days of initiation of Anti Tuberculosis Therapy (ATT)] were included in the PTB group. Patients with prior history of COVID-19 or immunocompromised conditions were excluded from this group. Newly diagnosed adult smear positive PTB patients in the age group of 18–60 yrs (within three days of initiation of ATT), with documented record of COVID-19 infection and positivity for SARS-CoV-2 IgG were included in the SARS-CoV-2 IgG+ve PTB group. Patients with prior history of immunocompromised disorders were excluded from this group also. For both the disease groups positivity for Mtb in sputum smear/NAAT and chest X-ray suggestive of TB were considered as the main criteria for inclusion. Healthy household contacts of patients attending the study sites in the age group of 18–60 yrs were included as healthy volunteers. Volunteers having a prior history of TB, COVID-19 (SARS-CoV-2 IgG positivity), diabetes or immunocompromised conditions, recent history of fever and/or cold, were also excluded. Pregnant and lactating women were not included for the study. The demographic profile and clinical features of the study participants and their signs/symptoms percentage are given in **Tables 1A**, **B**. A simple flow chart giving an overview of the study is given in **Figure 1**.

**Table 1A:** Demographics and clinical features of the study individuals.

Demographic details	Healthy volunteers	PTB patients	SARS-CoV-2 IgG+ve PTB patients	P value
No. of individuals	43	40	18	
Gender (M/F)	22/21	28/12	14/4	0.08
Age- Mean (range)	35 (24-60) yrs	43 (20-58) yrs	43 (21-58) yrs	
Smear grade				
Scanty/1+/2+/3+	NA	15/7/6/12	7/4/1/6	0.77
X-ray Cavitation				
Non cavitary/Cavitary	NA	15/20	8/9	0.99

Note: Fisher Exact test was used to check the significance.

X-rays of 6 participants were unavailable.

**Table 1B:** signs & symptoms of the study individuals.

Signs and symptoms	PTB patients (n=40)	SARS-CoV-2 IgG+ve PTB patients (n=18)	P value
Cough (%)	39 (97.5)	17 (94.44)	0.53
Fever (%)	24 (60)	12 (66.66)	0.77
Hemoptysis (%)	6 (15)	4 (22.22)	0.48
Dyspnea (%)	5 (12.5)	4 (22.22)	0.43
Weight loss (%)	31 (77.5)	13 (72.22)	0.74
Loss of apetite (%)	26 (65)	9 (50)	0.38

Note: Fisher Exact test was used to check the significance.

The study was carried out following Good Clinical Laboratory Practice (GCLP) guidelines. A trained phlebotomist collected 12 mL of whole blood in plain (biochemical testing and SARS-CoV-2 IgG testing), EDTA (hematological tests) and heparin (NET parameters) vacutainers from willing participants after obtaining written informed consent. Care was taken to avoid hemolysis during blood collection. Blood samples were stored and transported at room temperature, and processed for biochemical and hematological analyses within three hours. For measuring NET parameters, plasma was isolated from EDTA-treated whole blood and stored at -80°C until further assays. All quality control measures including instrument calibration, positive/negative controls and duplicate sampling were followed in order to get accurate and reliable data.

### 2.2 Evaluation of radiological parameters

The severity of the disease was evaluated through X-ray assessment performed in a blinded manner by two independent radiologists who were also unaware of the results of immunological or biochemical assays. Briefly, the type of lesions were scored as cavitray/non cavitary (14). In each X-ray category the number of cases in PTB and SARS-CoV-2 IgG+ve PTB were enumerated as shown in **Table 1A**. For six participants, the X-rays were unavailable and hence they were not included for this analysis.

### 2.3 Sputum preparation for acid-fast bacilli staining

Two sputum (spot and early morning) samples were collected from each study participant following National Tuberculosis Elimination Programme (NTEP) guidelines (15). Modified Petroff’s method (16) was used for sample decontamination and further diagnosis was made based on sputum microscopy by Zeihl–Neelsen staining. The presence of *Mycobacterium tuberculosis* was graded as low (scanty/1+) and high (2+/3+) smear grade as given in **Table 1a**. Smear grading was done following the grading criteria given in **Supplementary Table S1**.

### 2.4 Hematology assay

Complete blood count was determined from EDTA-treated whole blood using the BC-5150 Analyser (Mindray Global, China). Subsequently, plasma was isolated and stored at -80°C until further use. All the evaluated hematological parameters are given in **Supplementary Table S2**. Since this study is focused on neutrophils, we have presented only neutrophil counts in our results. Neutropenia was defined as neutrophils <44% and <42% in females and males respectively. On the other hand, neutrophilia was defined as neutrophils >75% and >74% in females and males respectively (17).

### 2.5 Anti-SARS-CoV-2 IgG assay

Anti-SARS-CoV-2 IgG CLIA was done using the SARS-CoV-2 IgG Assay kit (Siemens Healthineers Pvt Ltd.) following manufacturer’s instructions to determine past COVID -19 infection.

### 2.6 Biochemical assays

Biochemical parameters like lactate dehydrogenase (LDH), C-reactive protein (CRP), D-dimer and ferritin were measured using commercial reagents from Beckman Coulter Pvt Ltd. For this purpose serum was isolated from whole blood by centrifugation at 3500rpm for 10 minutes. The biochemical assays were run on the AU 480 Clinical Chemistry Analyser (Beckman Coulter, US) using freshly prepared serum samples. The quality of assay performance was periodically monitored using internal and external quality assurance procedures.

### 2.7 Enzyme linked immuno sorbent assay

NET markers like elastase, Myeloperoxidase (MPO) and Citrullinated Histone H3 and the cytokine IP10 were measured in stored plasma samples. MPO (R&D systems Cat# DMYE00B), elastase (R&D systems Cat# DY9167-05), Citrullinated Histone H3 (Cayman Chemicals Cat# 501620) & IP10 (BD Biosciences Cat#550926) were measured using commercial ELISA kits according to manufacturers’ instructions. In brief, pre- coated plates were used. Initially standards/samples were added and incubated at room temperature (RT) for 2 hours. Then the plates were washed and HRP conjugate was added and incubated for 1 hour. The plates were again washed and 3,3′,5,5′-Tetramethylbenzidine (TMB) substrate was added. After color development, further reaction was stopped by adding 2N H_2_SO_4_. The plates were read at 450nm/570nm in an iMark Microplate reader (BIO-RAD, USA). Concentrations were expressed in pg/mL. The sensitivity of detection for various parameters analyzed are provided in **Supplementary Table S3**.

### 2.8 Statistical analysis

GraphPad prism software (V10.4.1; GraphPad Software, Inc., San Diego, CA, USA) was used to perform all statistical analyses. Shapiro Wilk test was used to determine the normality of data. Statistical significance between study groups was evaluated using the Mann-Whitney U test for all parameters, except for MPO. Since MPO levels followed normal distribution, Student’s t-test was used. A value of P<0.05 was considered as statistically significant. The elastase levels were also compared between two different treatment outcomes- treatment success and relapse. Treatment success was defined as microbiologically confirmed TB patient at the beginning of treatment who is smear negative at the end of complete treatment. Relapse was defined as TB Patient previously declared as successfully treated and subsequently found to be microbiologically confirmed TB. Spearman’s rank correlation coefficient was used to assess relationships between hematological, biochemical, and NET parameters across the disease groups. A positive or negative value of 0.5 was considered as meaningful correlation. In order to understand the influence of confounding variables, logistic regression analysis was done to determine statistical significance of elastase levels between the disease groups using SPSS software (v.25).

## 3 Results

### 3.1 Neutrophilia is prevalent in PTB

As neutrophils are the primary contributors to NET formation we evaluated their counts in our study groups. 55% of recruits in the PTB group had neutrophilia compared to 78% in the SARS-CoV-2 IgG+ve PTB group. Neutropenia did not occur in any of the groups (**Table 2**). We found that the PTB group had a greater rate of neutrophilia (55%) than our previous observation (24%) (18).

**Table 2:** Neutrophil counts in the three different study groups.

Neutrophil percentage	Healthy (n=43) N (%)	PTB (n=40) N (%)	SARS-CoV-2 IgG+ve PTB (n=18) N (%)
Neutropenia (F<44%, M<42%)	1 (2.3)	0 (0)	0 (0)
Neutrophilia (F>75%, M>74%)	1 (2.3)	22 (55)	14 (78)
Neutrophils within range	41 (95.4)	18 (45)	4 (22)

### 3.2 Levels of NET markers other than elastase released during tuberculosis is not impacted by prior COVID-19 infection

**Figure 2:**
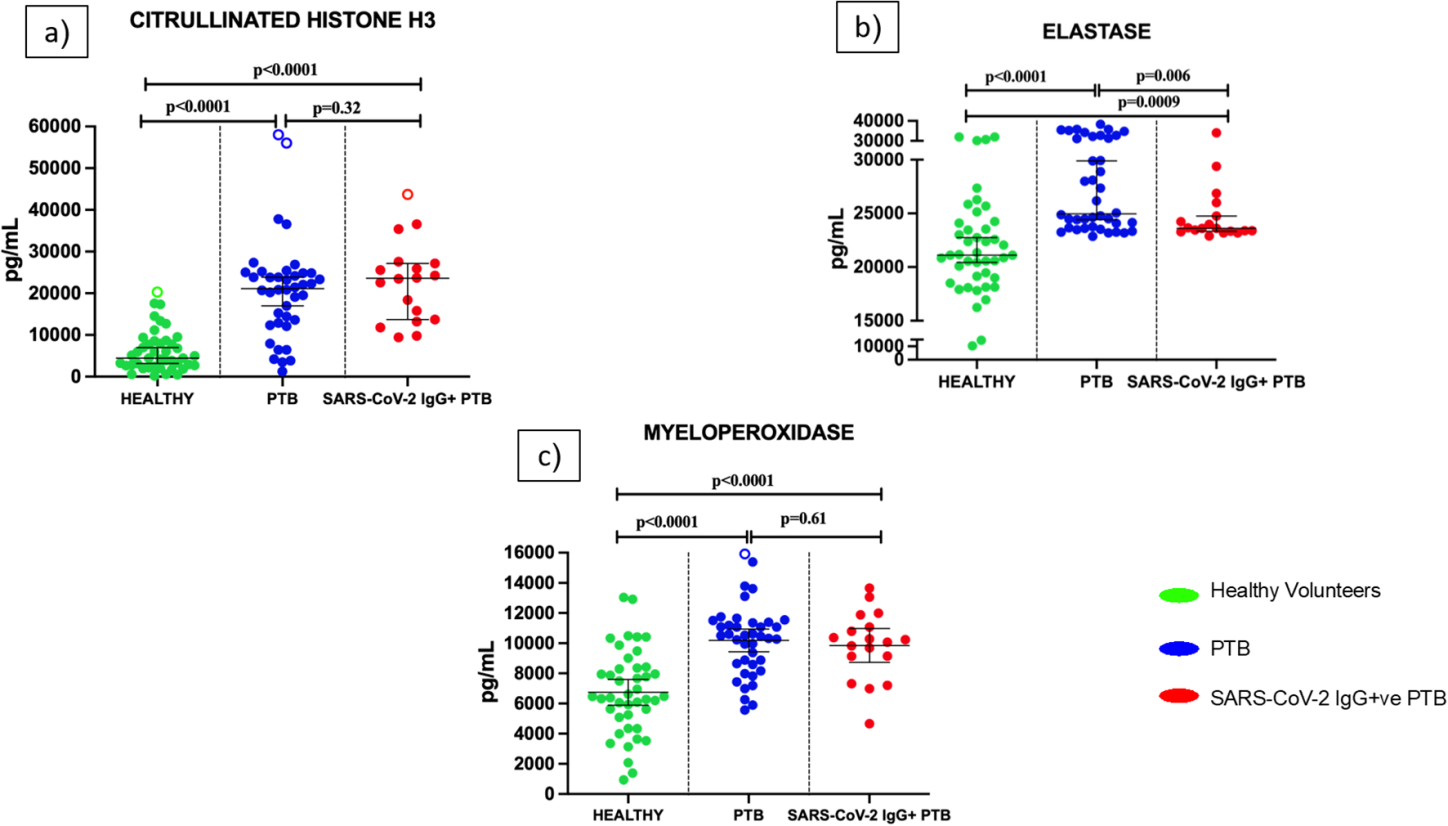
Levels of Neutrophil Extracellular Trap (NET) markers in the plasma of healthy volunteers, PTB and SARS-CoV-2 IgG+ve PTB participants: ELISA was done to measure the levels of Citrullinated Histone H3 **(a)**, elastase **(b)** and myeloperoxidase **(c)** in the plasma of Healthy (n=43), PTB (n=40) and SARS-CoV-2 IgG+ve PTB (n=18) participants. The P values were calculated using Mann-Whitney U test for Citrullinated Histone H3 and elastase and Students t-test for myeloperoxidase. The results for Citrullinated Histone H3 and elastase are shown as scattered dot plots with lines at median and 95% CI. The results for MPO are shown as scattered dot plots with lines at mean and 95% CI. Open circles represent outliers (larger than the upper quartile plus 1.5 times the interquartile range).

As shown in **Figure 2**, we measured the NET markers Citrullinated Histone H3 (a), elastase (b) and MPO (c) in plasma of healthy volunteers, PTB and SARS-CoV-2 IgG+ve PTB individuals. Levels of all three NET markers— Citrullinated Histone H3, elastase, and MPO—were significantly higher in both PTB and SARS-CoV-2 IgG+ve PTB groups compared to healthy controls (all p<0.001). However, when comparing PTB to SARS-CoV-2 IgG+ve PTB, no significant differences were observed for Citrullinated Histone H3 (p=0.32) or MPO (p=0.060), while elastase was significantly higher in the PTB group (median: 24,955 pg/mL; IQR: 23,679–31,958) compared to the SARS-CoV-2 IgG+ve PTB group (median: 23,608 pg/mL; IQR: 23,324–25,067; p=0.006). We further analyzed this difference in elastase levels across disease groups using a generalized linear model (GLM) with binary logistic regression. After adjusting for multiple confounding variables like age, diabetic status, smear status and lung involvement, elastase retained its statistical significance (p=0.030) between PTB and SARS-CoV-2 IgG+ve PTB groups (**Supplementary Table S4**).

### 3.3 Diabetes in the SARS-CoV-2 IgG+ve PTB group influences elastase levels

**Figure 3:**
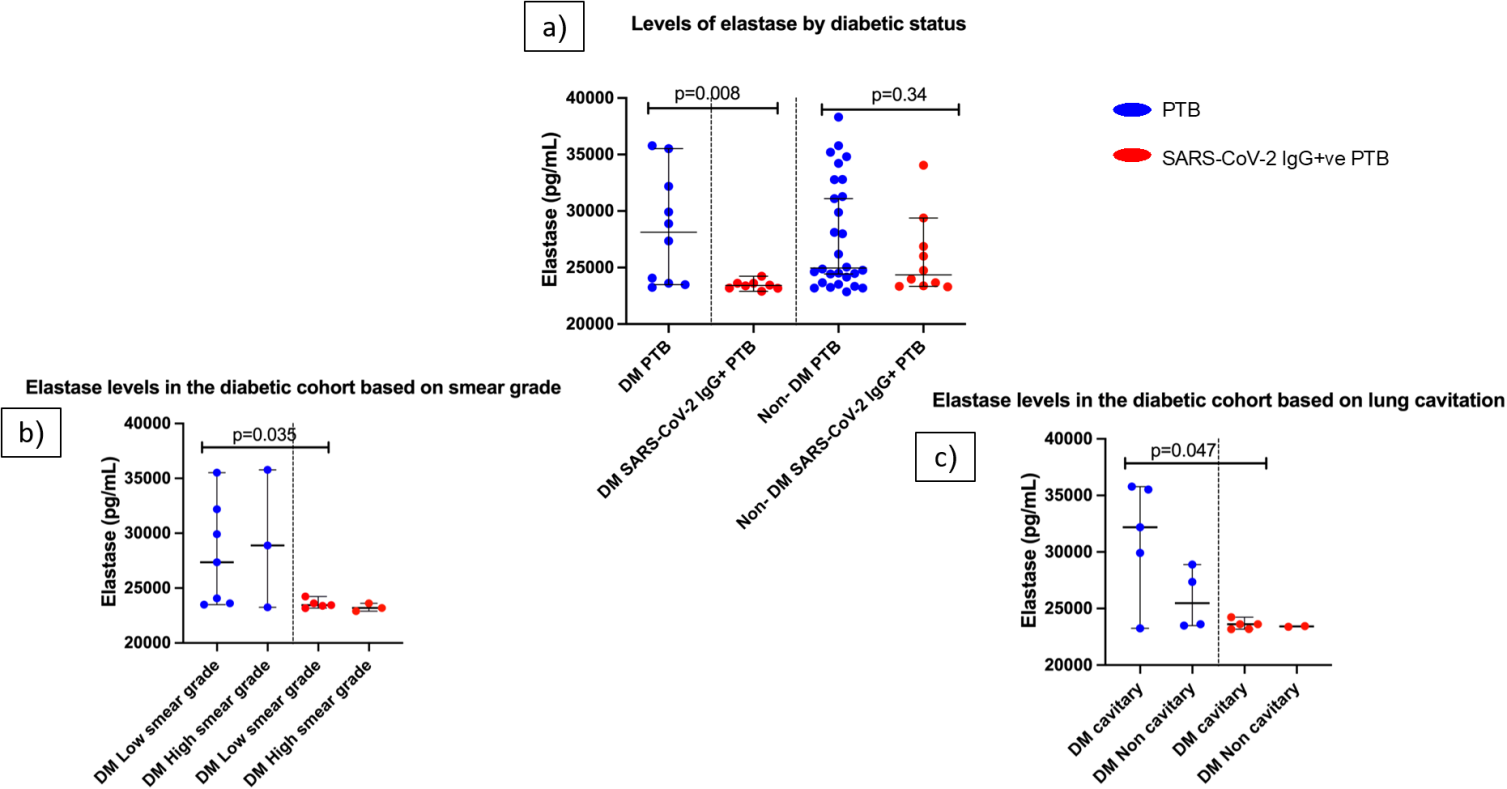
Influence of diabetes on elastase levels in PTB and SARS-CoV-2 IgG+ve PTB disease groups: **(a)** shows the elastase levels in DM PTB (n=10), DM SARS-CoV-2 IgG+ve PTB (n=8), non-DM PTB (n=28) and non- DM SARS-CoV-2 IgG+ve PTB (n=10) groups. **(b, c)** show the elastase levels in diabetic cohort of PTB and SARS-CoV-2 IgG+ve PTB groups based on smear grades (low/high) and lung cavitation (cavitary/non cavitary] respectively. The P values were calculated using Mann-Whitney U test. The data are shown as scattered dot plots with lines at median and 95% CI and each circle representing a single individual.

The SAR-CoV-2 IgG+ve group had a higher percentage of diabetic patients (44.4%) compared to the PTB group (26%) (**Supplementary Table S5**). To evaluate the influence of diabetes on elastase levels, we segregated the patients in each group as diabetic (under diabetic treatment for at least one year) and non-diabetic. The diabetic cohort of the SARS-CoV-2 IgG+ve PTB group had significantly lower levels of elastase (p=0.008) than the diabetic cohort of the PTB group (**Figure 3a**).

The clinical characteristics of the diabetes cohort in both disease groups were then examined. For this purpose, smear grades were classified as low (scanty/1+) and high (2+/3+). Chest X- rays were divided into those with and without cavitary lesions. Even though there was no statistically significant difference in the participant distribution across these clinical categories (**Supplementary Tables S6**, **S7**), SARS-CoV-2 IgG+ PTB group had significantly decreased elastase levels in the low smear grade category compared to PTB group (p=0.035) (**Figure 3b**). In the high smear grade category, there was no significant difference between the groups. Considering X- rays, elastase levels in the cavitary group of PTB was significantly higher than the SARS-CoV-2 IgG+ve PTB group (p=0.047) whereas the non-cavitary group did not exhibit statistical difference (**Figure 3c**). These results emphasize that even within same clinical category between the two disease groups, there exists difference in elastase levels when they are diabetics. Elastase concentration as median and IQR for this sub group analysis is available in **Supplementary Table S8**.

### 3.4 Elastase level reflects outcome in PTB patients with prior history of COVID-19 infection

**Figure 4:**
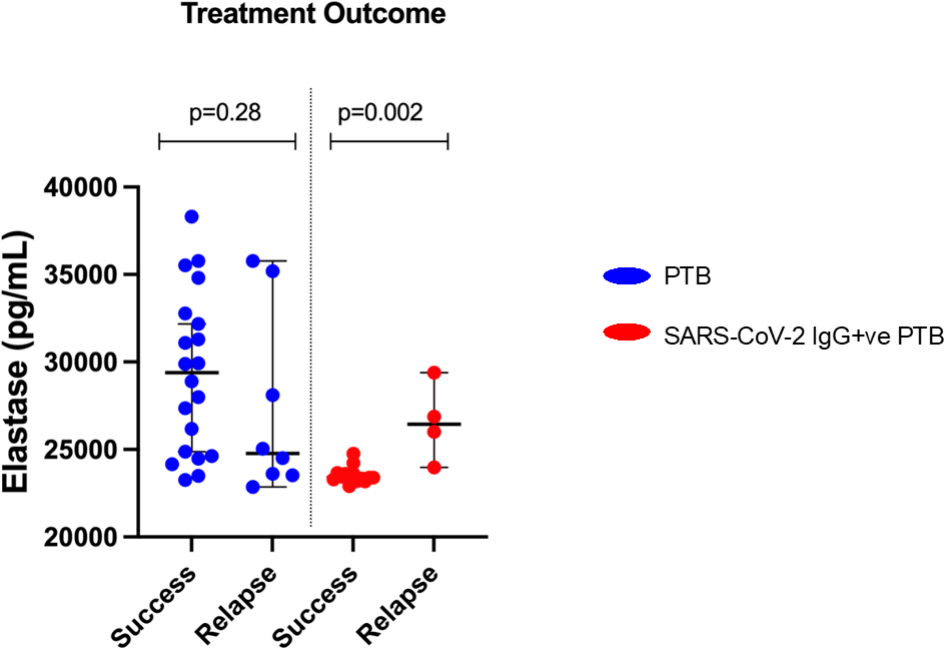
Elastase levels and difference in treatment outcomes between PTB and SARS-CoV-2 IgG+ve PTB groups: Plasma elastase was measured by ELISA in PTB and SARS-CoV-2 IgG+ve PTB groups. The treatment outcome of PTB (n=28) and SARS-CoV-2 IgG+ve PTB (n=17) groups are plotted against elastase levels and represented as scattered dot plots with lines at median and 95% CI. The P values were calculated using Mann-Whitney U test.

As shown in **Figure 4**, in the SARS-CoV-2 IgG+ve PTB group, participants who had successful treatment had significantly lower levels of elastase (p=0.002) compared to those who had relapse. In the PTB group, elastase levels did not differ between different treatment outcomes (success/relapse).

### 3.5 Inflammatory parameters in PTB patients are not influenced by prior history of COVID-19 infection

**Figure 5:**
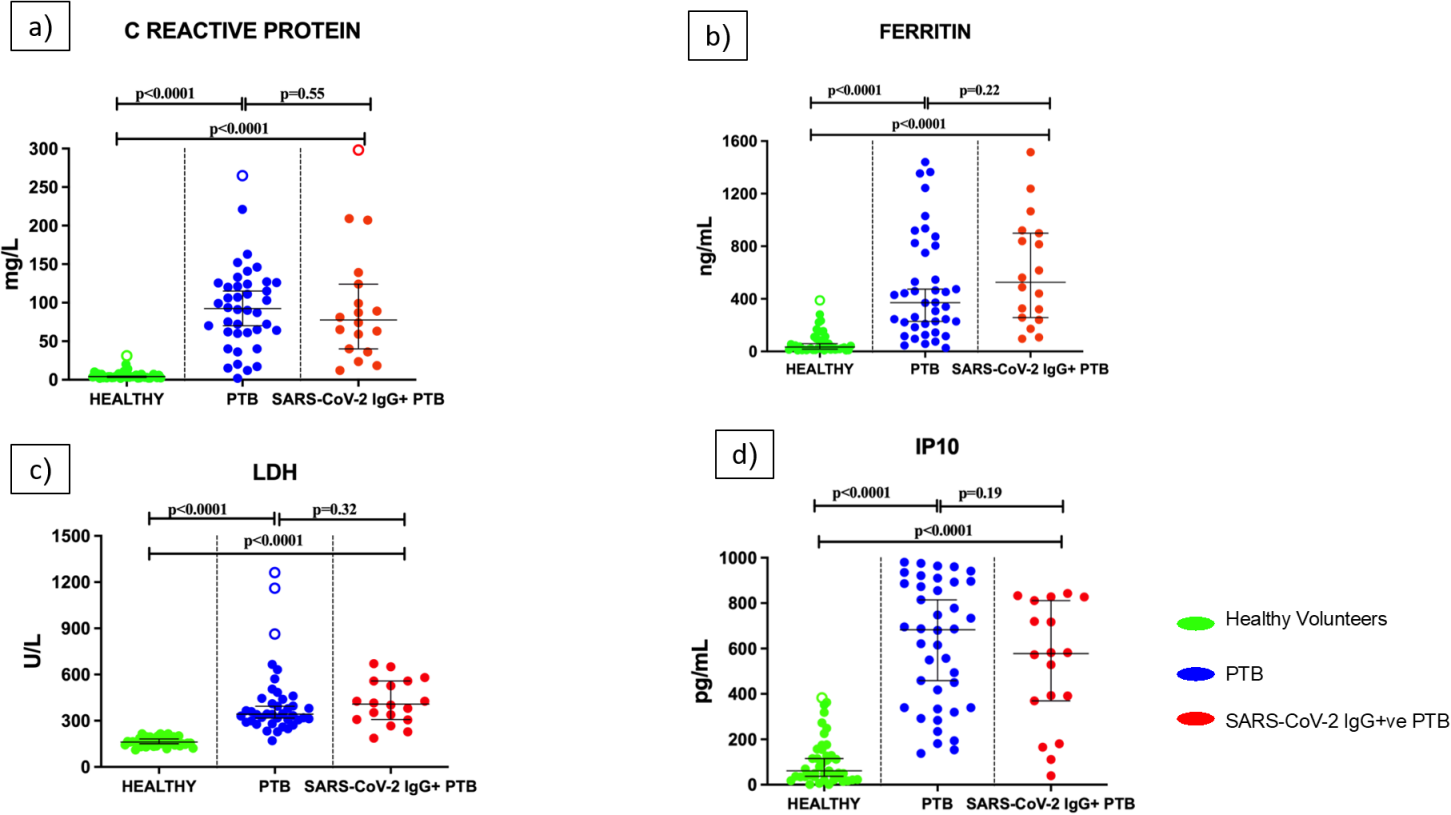
Levels of inflammatory parameters in the serum/plasma of healthy volunteers, PTB and SARS-CoV-2 IgG+ve PTB participants: C-reactive protein (mg/L), Ferritin (ng/mL), Lactate Dehydrogenase (U/L) and IP10 (pg/mL) were measured in Healthy (n=43), PTB (n=40) and SARS-CoV-2 IgG+ve PTB (n=18) groups as shown in **(a-d)**. The P values were calculated using Mann-Whitney U test. The results are shown as scattered dot plots with lines at median and 95% CI. Open circles represent outliers (larger than the upper quartile plus 1.5 times the interquartile range).

As illustrated in **Figures 5a-d**, the concentration of inflammatory markers- CRP, ferritin, LDH and IP10 were significantly higher in PTB and SARS-CoV-2 IgG+ve PTB groups compared to the healthy volunteers (all p <0.0001). No significant differences were observed for these analytes between the PTB and SARS-CoV-2 IgG+ve PTB groups.

### 3.6 Triple pathway correlation

**Figure 6:**
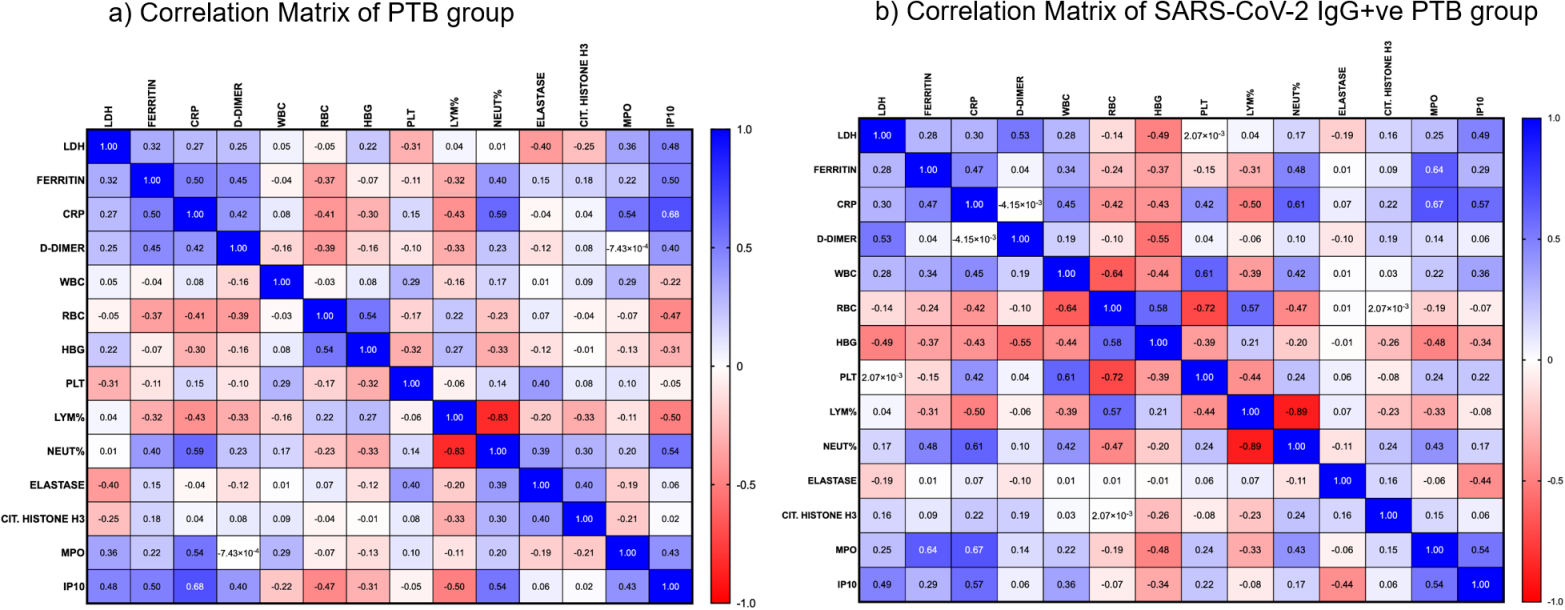
Correlation analysis between hematological, biochemical and NET markers: **(a, b)** show the strength of association between various hematological, biochemical and NET markers in the PTB and SARS-CoV-2 IgG+ve PTB groups respectively. Spearman’s correlation coefficients are visualized by color intensity.

A correlation matrix was constructed for PTB and SARS-CoV-2 IgG+ve PTB groups to understand the existing association between the hematological, biochemical and NET parameters. As shown in **Figures 6a, b**, there was a moderate positive correlation between the following parameters in the PTB group: CRP and MPO (r= 0.54, p=0.0003); CRP and ferritin (r= 0.50, p=0.001); CRP and IP10 (r= 0.68, p<0.0001); CRP and neutrophil percentage (r= 0.59, p<0.0001); IP10 and ferritin (r= 0.50, p=0.002); IP10 and neutrophil percentage (r= 0.54, p=0.002). There was a moderate positive correlation between the following parameters in the SARS-CoV-2 IgG+ve PTB group: MPO and ferritin (r= 0.64, p=0.004); MPO and IP10 (r= 0.54, p=0.033); MPO and CRP (r= 0.67, p=0.002); neutrophil percentage and CRP (r= 0.61, p=0.007); IP10 and CRP (r= 0.57, p=0.022). Lymphocyte percentage had a moderate negative correlation with IP10 (r= -0.50, p=0.001) in the PTB group and CRP (r= -0.50, p=0.036) in the SARS-CoV-2 IgG+ve PTB group.

## 4 Discussion

Neutrophils continuously patrol the vasculature, monitoring for signals of infection or inflammation (19). One of the neutrophil’s defense mechanism is the development of NETs, where they extrude the DNA embedded with antimicrobial enzymes to entrap and kill microbes (20). This process has been well documented in various complications of COVID-19 (12). Biochemical analytes like LDH, D-dimer, CRP and ferritin have also been established as useful tools to assess severity and prognosis of COVID-19 (21, 22). However, little is known about the function of NETs in the diagnosis and prognosis of tuberculosis, as well as how they relate to other analytes. Earlier investigations have documented that breakdown of intrinsic regulation of NET-driven tissue damage happens in severe TB cases with increased levels of Citrullinated Histone H3 (23). Also, suppression of NETs has been reported to be protective in various animal models of influenza-associated ARDS (24). Therefore, we were interested to evaluate the levels of NET markers in PTB in relation to a prior COVID-19 infection. Additionally, we assessed the levels of IP10, a potential biomarker for TB diagnosis and disease progression (25–27) and a component of the cytokine storm during COVID-19 (28). We tried to find its association with NET markers in order to identify its role in influencing NET formation during tuberculosis, post COVID-19 infection.

Critical COVID-19 cases are found to have the greatest levels of NET markers, indicating that NETs are correlated with the severity of the illness (29, 30). Our finding that MPO and Citrullinated Histone H3 levels are similar during tuberculosis regardless of a prior history of COVID-19 suggests that the NET response mediated by these molecules during SARS-CoV-2 infection may have decreased after recovery and therefore does not affect NET levels during subsequent TB disease. However, this was not the case for elastase levels as we observed significantly decreased levels in the tuberculosis group with SARS-CoV-2 IgG positivity. Elastase is reported to be involved in lung tissue destruction in chronic obstructive pulmonary disease (31, 32) and severe multi-organ and systemic symptoms in COVID-19 (33). Additionally, elastase is associated with computer tomographic (CT) evidence of bronchiectasis in children with Cystic Fibrosis (34). But in tuberculosis, despite the fact that elastase levels are reported to be higher in humans (13), there are no reports to link elastase to pathology. Meanwhile, elastase has been demonstrated to reduce early mycobacterial outgrowth during the acute phase of mycobacterial infection in animal models (35). Hence, more research is required to completely comprehend the implications of the lowered elastase levels during TB disease following COVID-19 infection.

Given the low and intriguing levels of elastase seen in the PTB SARS-CoV-2 IgG+ve group, more investigation and confirmation are required to identify the underlying causes. Therefore, we looked at whether elastase levels would be affected by the varying distribution of confounding factors such as smear grade and/or the existence of cavities (X-ray) between the disease groups. Nonetheless, there was a similar distribution of participants in both groups with cavitary X-rays and smear grades. The potential impact of previous COVID-19 infection (as confirmed by SARS-CoV-2 IgG levels) on elastase levels was then examined. This was accomplished by comparing the levels of elastase and SARS-CoV-2 IgG in the two groups (data not shown). Nevertheless, there was no association between the antibody levels and elastase. Our hematological data was analyzed to rule out the notion that the SARS-CoV-2 IgG+ve group produced less elastase because they had fewer neutrophils. According to our results, this group experienced neutrophilia, which is a well-established symptom of COVID-19 (11, 36). Since we recruited participants in both groups during the second wave of COVID-19, it is believed that the heterogeneity of SARS-CoV-2 did not significantly affect the observed values of many parameters, including elastase. However, transmission dynamics may differ amongst individuals.

We looked for other variables that could have affected the two PTB groups’ elastase levels. We came across literature suggesting that elastase plays a substantial role in diabetes associated complications including diabetic retinopathy (37) and concurrent myocardial infarctions (38). Nonetheless, patients with type 2 diabetes mellitus per se (39) and those treated with metformin, the most commonly used oral hypoglycemic medication (40), had lower elastase levels. Therefore, both disease groups were subjected to a subgroup analysis based on the presence or absence of diabetes. We assume that the elastase levels in the SARS-CoV-2 IgG+ PTB group have been impacted by the cohort’s diabetes status, as seen in **Figure 3**, where the diabetic arm of the group is observed to produce lower elastase levels. However, this observation is entirely valid only in the SARS-CoV-2 IgG+ PTB group but not in the PTB group, where diabetes was not shown to alter elastase secretion. We hypothesize that this discovery stems from metformin’s anti-NET activity, which may increase or contribute to its advantages for COVID-19 patients, as discussed by Thierry, 2024 (41). Interestingly, while the diabetic subgroup of the SARS-CoV-2 IgG+ PTB participants experienced successful treatment outcomes (Success: relapse; 8:0), the non-DM cohort had a mixture of outcomes (Success: relapse; 5:4). Therefore our data suggests that when TB develops after SARS-CoV-2 infection, diabetes (or its treatment) might be influential. The small sample size, however, limits our ability to validate our hypothesis; future large cohort studies may provide a more accurate picture.

Since it has been previously established that general biochemical parameters during tuberculosis followed by prior SARS-CoV-2 infection does not vary significantly from tuberculosis alone (42), we wanted to expand this finding to biochemical indicators of severe COVID-19 such as ferritin, CRP, LDH and IP10. Regardless of prior COVID-19 infection, we found that these inflammatory markers remain unchanged after contracting TB disease. For the first time, we report this in relation to the incidence of TB in previous COVID-19-infected patients, even though these indicators have been extensively studied in the context of COVID-19.

In order to better understand the biological processes involved in the disease conditions under investigation, we also looked at the association between inflammatory, biochemical and NET markers. Although the levels of inflammatory molecules and NET markers (Citrullinated Histone H3 and MPO) were similar in TB patients with and without previous COVID-19 infection, the correlation matrix revealed different patterns in both groups This observation suggests that COVID-19 sequelae cause biological differences in NETosis and biochemical analytes. Considering the positive association we observed between MPO and CRP, it is plausible that MPO serves as an indirect measure of inflammation. Also, when combined with CRP, it could be a useful predictor of the risk of death from cardiovascular disease (43). Expanding on the observation made during COVID-19 infection, a positive correlation between MPO and ferritin during tuberculosis after COVID-19 infection suggests a role for ferritin-mediated NET formation (44, 45). Our examination of inflammatory analytes showed that in both groups, CRP and IP10 had notable correlations with other parameters. Regardless of a patient’s prior history of COVID-19, the discovery that IP10 levels during tuberculosis positively correlate with other hematological and biochemical markers highlights the significance of cytokine-mediated responses in the pathophysiology of TB. It is well established that platelets and RBCs play a crucial role in thrombin generation (46). Only in the PTB group did we observe a negative correlation between them. This suggests the possibility of a different coagulation pattern in the PTB group without a history of COVID-19 infection.

## 5 Limitations

One of the limitations of our study is the small sample size. In particular, if we had been able to expand the sample size and conduct a longitudinal follow-up with the individuals, our hypothesis that diabetes influences elastase levels in the PTB group with previous COVID-19 infection would have been validated. To support this assumption, we plan to conduct additional studies including a larger cohort of people with diabetes. Additionally, in the SARS-CoV-2 IgG+ve group, we were unable to track the exact time and severity of SARS-CoV-2 infection. This information would have been valuable in determining whether these confounders influence NET and inflammatory marker levels.

## 6 Conclusion

To the best of our knowledge, this is the first report showing that prior COVID-19 infection affects elastase levels while having no effect on MPO, Citrullinated Histone H3, LDH, ferritin or CRP production in TB patients. Our findings need to be validated using longitudinal studies to determine how NETosis resolves with anti-tuberculosis therapy in order to plan suitable future host-directed therapies.

## Data Availability

The raw data supporting the conclusions of this article will be made available by the authors, without undue reservation. Further inquiries regarding data availability can be directed to the corresponding author/s.
