# Supplementary material for: Previous COVID-19 infection significantly reduces elastase levels in newly diagnosed pulmonary tuberculosis patients
Source: Front Immunol. 2025 Jul 31;16:1586789. doi: 10.3389/fimmu.2025.1586789 (PMC12350145; doi:10.3389/fimmu.2025.1586789)
Supplement: Supplementary file 1 [file DataSheet1.pdf]

Table S1: Grading criteria for Acid-fast Bacillus (AFB) smear microscopy: The smear grading criteria followed to distinguish the smear grade is given in the following table.

Examination	Grading	No. of fields to be examined
10 AFB per oil immersion field	3+	20
1-10 AFB per oil immersion field	2+	50
10-99 AFB per 100 oil immersion fields	1+	100
1-9 AFB per 100 oil immersion fields	Scanty	100
No AFB per 100 oil immersion fields	Negative	100

Table S2: Hematological parameters observed in the study groups: Basic hematological parameters were measured in healthy, PTB and SARS-CoV-2 IgG+ve PTB groups using BC5150 analyser and their mean and SD values are given in this table.

Hematological Parameters	Units	Healthy Volunteers (n=43)	PTB participants (n=40)	SARS-CoV-2 IgG+ve PTB participants (n=18)
		Mean (SD)	Mean (SD)	Mean (SD)
Hemoglobin	g/dL	13.73 (2.40)	11.83 (1.92)	12.25 (2.12)
Platelets	10^3 cells/μL	300.0 (86.27)	408.40 (167.0)	400.60 (121.8)
Red Blood Cells	10^6 cells /μL	4.926 (0.56)	4.57 (0.81)	4.56 (0.69)
White Blood Cells	10^3 cells /μL	7.64 (2.19)	11.55 (2.99)	11.80 (3.10)
Lymphocyte	%	33.35 (6.34)	16.48 (5.54)	13.34 (5.88)
Neutrophil	%	57.62 (10.36)	75.55 (6.28)	78.63 (8.28)

Table S3: Sensitivity of parameters used: The given table shows the sensitivity of each parameter measured by ELISA from the plasma of healthy volunteers, PTB and SARS-CoV-2 IgG+ve PTB participants.

Analyte	pg/mL
Citrullinated Histone H3[1]	150
Elastase[2]	46.9
Myeloperoxidase[2]	62
IP10[3]	1.67

[1] Commercial ELISA kits from Cayman Chemical, USA

[2] Commercial ELISA kits from R&D systems, USA

[3] Commercial ELISA kits from BD Biosciences, USA

Table S4: Regression Analysis: The given table shows the significance of elastase between PTB and SARS-CoV-2 IgG+ve PTB groups after adjusting for confounding variables using binary logistic regression.

Variables	B	95% confidence interval		p value
		lower	upper	
**Elastase**	**-0.000216138**	**-0.0004119**	**-0.0000204**	**0.030**
Age	-0.008595703	-0.0678129	0.0506215	0.77
Smear status	-0.108276156	-1.4644344	1.2478821	0.87
Diabetic status	-0.573380236	-1.9900218	0.8432613	0.42
Lung involvement	-0.420053718	-1.8073689	0.9672614	0.55

Table S5: Distribution of diabetic population in PTB and SARS-CoV-2 IgG+ve PTB groups: The given table shows the diabetic status of the study participants in PTB and SARS CoV IgG+ PTB groups. Two participants in the PTB group whose diabetic status were unknown was not considered for this analysis.

Diabetic Status	PTB (n= 38)	SARS CoV IgG+ PTB (n=18)
	n (%)	n (%)
No	28 (74%)	10 (55.6%)
Yes	10 (26%)	8 (44.4%)

Table S6: Distribution of smear grades in the diabetic and non-diabetic cohorts of PTB and SARS-CoV-2 IgG+ve PTB groups: The given table shows the distribution of smear grades of participants according to their diabetic status respectively. Two participants in the PTB group whose diabetic status were unknown was not considered for this analysis.

Diabetic Participants	PTB (n=10)	SARS CoV IgG+ PTB (n=8)	p value
	Low smear grade (n=7)<br>High smear grade (n=3)	Low smear grade (n=5)<br>High smear grade (n=3)	0.99
Non-Diabetic Participants	PTB (n=28)	SARS CoV IgG+ PTB (n=10)	0.71
	Low smear grade (n=13)<br>High smear grade (n=15)	Low smear grade (n=6)<br>High smear grade (n=4)	

* Fisher's exact test was used to compute significance.

Table S7: Chest X-ray Cavitation in the diabetic and non-diabetic cohorts of PTB and SARS-CoV-2 IgG+ve PTB groups: The given table shows the distribution of X-ray cavitation of participants according to their diabetic status respectively. Two participants in the PTB group whose diabetic status were unknown was not considered for this analysis.

Diabetic Participants	PTB (n=9)	SARS CoV IgG+ PTB (n=7)	p value
	Non Cavitary (n=4)<br>Cavitary (n=5)	Non Cavitary (n=2)<br>Cavitary (n=5)	0.63
Non- Diabetic Participants	PTB (n=26)	SARS CoV IgG+ PTB (n=10)	0.28
	Non Cavitary (n=10)<br>Cavitary (n=16)	Non Cavitary (n=6)<br>Cavitary (n=4)	

*X-rays of 6 participants were unavailable
*Fisher's exact test was used to compute significance.

Table S8: Elastase concentration shown as median and interquartile range for different sub group analyses within the disease groups

Groups		Elastase levels as Median (IQR)	p value
DM PTB (n=10)		28,123 (23,580- 33,017)	0.008
DM SARS-CoV-2 IgG+ve PTB (n=8)		23,421 (23,180-23,608)	
Low smear grade	DM PTB (n=7)	27,353 (23,608-32,183)	0.035
	DM SARS-CoV-2 IgG+ve PTB (n=5)	23,449 (23,285-23,920)	
Cavitary	DM PTB (n=5)	32,183 (26,588-35,648)	0.047
	DM SARS-CoV-2 IgG+ve PTB (n=5)	23,608 (23,182-23,920)	
SARS-CoV-2 IgG+ve PTB	Treatment success (n=13)	23,392 (23,239- 23,631)	0.002
	Relapse (n=4)	26,439 (24,480- 28,761)	
